# Teneurins: Domain Architecture, Evolutionary Origins, and Patterns of Expression

**DOI:** 10.3389/fnins.2018.00938

**Published:** 2018-12-11

**Authors:** Richard P. Tucker

**Affiliations:** Department of Cell Biology and Human Anatomy, University of California at Davis, Davis, CA, United States

**Keywords:** ABC toxin, brain, development, horizontal gene transfer, odz, teneurin, YD protein

## Abstract

Disruption of teneurin expression results in abnormal neural networks, but just how teneurins support the development of the central nervous system remains an area of active research. This review summarizes some of what we know about the functions of the various domains of teneurins, the possible evolution of teneurins from a bacterial toxin, and the intriguing patterns of teneurin expression. Teneurins are a family of type-2 transmembrane proteins. The N-terminal intracellular domain can be processed and localized to the nucleus, but the significance of this nuclear localization is unknown. The extracellular domain of teneurins is largely composed of tyrosine-aspartic acid repeats that fold into a hollow barrel, and the C-terminal domains of teneurins are stuffed, and least partly, into the barrel. A 6-bladed beta-propeller is found at the other end of the barrel. The same arrangement—6-bladed beta-propeller, tyrosine-aspartic acid repeat barrel, and the C-terminal domain inside the barrel—is seen in toxic proteins from bacteria, and there is evidence that teneurins may have evolved from a gene encoding a prokaryotic toxin via horizontal gene transfer into an ancestral choanoflagellate. Patterns of teneurin expression are often, but not always, complementary. In the central nervous system, where teneurins are best studied, interconnected populations of neurons often express the same teneurin. For example, in the chicken embryo neurons forming the tectofugal pathway express teneurin-1, whereas neurons forming the thalamofugal pathway express teneurin-2. In *Drosophila melanogaster, Caenorhabditis elegans*, zebrafish and mice, misexpression or knocking out teneurin expression leads to abnormal connections in the neural networks that normally express the relevant teneurin. Teneurins are also expressed in non-neuronal tissue during development, and in at least some regions the patterns of non-neuronal expression are also complementary. The function of teneurins outside the nervous system remains unclear.

## Introduction

**FIGURE 1:**
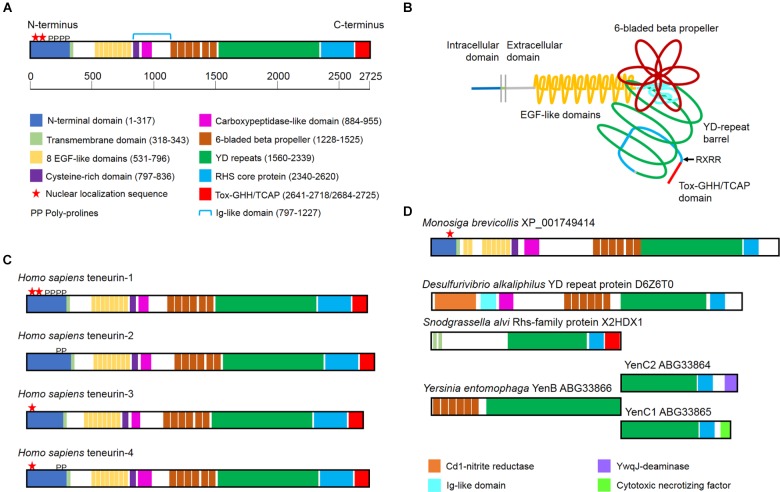
The domain organization of teneurins and teneurin-related YD proteins from prokaryotes. **(A)** The domain organization of a typical teneurin, human teneurin-1. A colored key and a scale bar indicating the amino acid positions from N-terminus to C-terminus is shown. The Ig-like domain, which includes both a conserved cysteine-rich domain and a carboxypeptidase-like domain, is indicated with the blue bracket. **(B)** A schematic illustrating the tertiary organization of a typical teneurin. Key features in the extracellular domain includes the tyrosine-aspartic acid (YD)-repeat barrel and the 6-bladed beta-propeller, which is exposed for protein–protein interactions. Current evidence indicates that the C-terminal Tox-GHH/TCAP domain is found outside the barrel, as is a conserved RxRR motif, which may represent a proteolytic cleavage site. **(C)** Most vertebrates have four teneurins numbered 1 through 4. The domain organization of the four teneurins from humans are illustrated. **(D)** A predicted teneurin is found in the genome of the choanoflagellate *Monosiga brevicollis*. The extracellular domain of this teneurin is most similar to the extracellular domains of bacterial toxins. Examples of the bacterial toxins are illustrated, as are UniProt ID and GenBank accession numbers.

Teneurins are type-2 transmembrane proteins with a variable N-terminal intracellular domain and a large, phylogenetically conserved extracellular domain. The extracellular domain features epidermal growth factor (EGF)-like domains, a 6-bladed beta-propeller composed of NHL repeats, tyrosine-aspartic acid (YD) repeats, a rearrangement hot spot (RHS) core protein domain and a C-terminal domain related to both GHH toxins and corticotropin-releasing factor (Figure 1A). The genomes of most vertebrates include four related teneurin genes encoding teneurins numbered 1 through 4 (Tucker et al., 2012). In *Drosophila melanogaster* there are two teneurins, ten-a and ten-m (Baumgartner et al., 1994; Levine et al., 1994), and in *Caenorhabditis elegans* there is a single teneurin, ten-1 (Drabikowski et al., 2005). This review will concentrate on what is known about the domain organization of the best studied teneurins, what can be inferred about their evolution from studies of extant genomes, and patterns of teneurin expression.

## Teneurin Domain Organization

### The Teneurin Intracellular Domain

The teneurin intracellular domain typically includes one or more proline-rich SH3-binding domain and one (or more) predicted nuclear localization sequence (Figure 1A). Yeast two-hybrid screens and co-immunoprecipitation experiments demonstrated that one of the SH3-binding domains from teneurin-1 binds CAP/ponsin (Nunes et al., 2005). CAP/ponsin, also known as sorbin, is a widely expressed adaptor protein involved in the organization of the cytoskeleton and growth factor-mediated signaling (Kioka et al., 2002). The intracellular domain of teneurin-1 also binds to MBD1, a methylated DNA binding protein (Nunes et al., 2005), but the biological significance of this interaction is unknown. When the intracellular domain is overexpressed in tissue culture cells it is found in the nucleus where it co-localizes with PML protein in nuclear bodies (Bagutti et al., 2003; Nunes et al., 2005). In chicken embryos antibodies to the intracellular domain of teneurin-1 often stain the cell nucleus in regions where antibodies to the extracellular domain stain the cell surface (Kenzelmann et al., 2008; Kenzelmann Broz et al., 2010), suggesting that teneurins may be processed so that the intracellular domain can be released for yet-to-be determined function in the nucleus. A likely site for proteolytic cleavage within the intracellular domain is the conserved basic sequence motif RKRK. When fibroblasts are transfected with native teneurin-1, antibodies to the intracellular domain of teneurin-1 stain the nucleus, but they do not stain the nucleus if the cells are transfected with a teneurin-1 following mutation of the basic motif to AAAA (Kenzelmann et al., 2008). The potential for this type of processing has recently been confirmed by others (Vysokov et al., 2016). Finally, there are many alternatively spliced variants of the intracellular domains of teneurins from chicken and human (Tucker et al., 2012), but the biological significance of these variants is unknown.

### EGF-Like Domains

Most teneurins have eight EGF-like domains starting approximately 200 amino acids C-terminal to their transmembrane domain (Tucker et al., 2012). The Basic Local Alignment Search Tool (BLAST) reveals that these domains, which have the conserved consensus sequence Ex_2_Cx(D/N)x_2_Dx(D/E)xDx_3_DCx_3_(D/E)CCx_4_Cx_5_C (where “x” is any amino acid), are most similar to those found in the tenascin family of extracellular matrix glycoproteins. This explains why teneurins were first identified in a low stringency screen of *Drosophila* DNA with a probe based on the EGF-like domains of chicken tenascin-C (Baumgartner and Chiquet-Ehrismann, 1993). The names given to the *Drosophila* teneurins, ten-a and ten-m, reflect this historical connection to tenascins. In turn, the name “teneurin” is a conflation of “ten-a/ten-m” and “neurons,” which are a major site of teneurin expression (Minet et al., 1999). Note that teneurins were discovered independently in *D. melanogaster* and named *odd Oz* (Levine et al., 1994), which accounts for the alternative name Odz for teneurins in the literature and in some genome search engines.

One well-established function of the teneurin EGF-like domains is to permit dimerization in cis. Most teneurin EGF-like domains have six cysteines that form three pairs of disulfide bonds. However, the second and fifth teneurin EGF-like domains have only five cysteine residues. The odd number allows cysteines in one teneurin to make disulfide bonds with cysteines in a neighboring teneurin, resulting in covalently linked side-by-side dimers (Oohashi et al., 1999; Feng et al., 2002). This explains the distinctive “pair of cherries” appearance of the extracellular domain of teneurins when viewed in the electron microscope after rotary shadowing: the stems are the attached EGF-like domains, and the cherries are the remaining C-terminal part of the extracellular domain (Feng et al., 2002).

### Beta-Propeller Domain

The central region of the teneurin extracellular domain was first predicted to fold like a beta-propeller (i.e., it contains a series of NHL repeats) in an early study of teneurin domain architecture (Tucker et al., 2012) and later demonstrated conclusively to be a 6-bladed beta-propeller by X-ray crystallography and cryoelectron microscopy (Jackson et al., 2018; Li et al., 2018). Beta-propellers are typically protein–protein interaction domains, and that appears to be the case with teneurins. HT1080 cells expressing the transmembrane and extracellular domains of teneurin-2 clump together in culture, but HT1080 cells expressing the transmembrane domain and a truncated extracellular domain that only includes the EGF-like domains do not (Rubin et al., 2002). The domain used for these teneurin–teneurin interactions was narrowed to the 6-bladed beta-propeller using atomic force microscopy while swapping and deleting the various teneurin extracellular domains that were expressed on the cell surface (Beckmann et al., 2013). This study also showed that the homotypic interactions between the beta-propellers of teneurin-1 were stronger than the heterotypic interactions between the beta-propellers of teneurin-1 and teneurin-2 (Beckmann et al., 2013). The beta-propeller domain of teneurin-1 seems to be critical for its function, as a mutation in this region leads to congenital anosmia in humans (Alkelai et al., 2016).

### YD Repeats and the RHS Core Protein Domain

Almost a third of the huge extracellular domain of teneurins is composed of over two dozen YD repeats. These repeats have the consensus sequence Gx_3-9_YxYDx_2_GR(L, I or V)x_3-10_G, where “x” is any amino acid (Minet et al., 1999; Minet and Chiquet-Ehrismann, 2000). The presence of YD repeats in teneurins was unexpected: prior to the sequencing of human and chicken teneurins YD repeats had only been identified in prokaryotic proteins. The potential function of the YD repeats became clear following the detailed description of a similar series of repeats found in a toxin from the bacterium *Yersinia entomophaga* using X-ray crystallography (Busby et al., 2013). The YD repeats in this bacterial toxin form a hollow barrel that is approximately 130 Å long and 50 Å wide [i.e., the approximate size of the “cherry” of the teneurin extracellular domain seen in the electron microscope (Feng et al., 2002)]. The RHS core protein domain forms a plug in the hollow end of the barrel. This bacterial YD repeat-containing protein also has a 6-bladed beta-propeller, and the beta-propeller is exposed to ligand binding at the N-terminal end of the barrel. The high degree of sequence similarity and domain architecture identity between the C-terminal half of the extracellular domains of teneurins and these YD repeat-containing proteins from bacteria strongly suggested that teneurins would fold in a similar way. This was recently confirmed by X-ray crystallography and cryoelectron microscopy with teneurin extracellular domains (Jackson et al., 2018; Li et al., 2018).

### C-Terminal Domain: A Toxin and a TCAP

Just C-terminal to the RHS core protein domain of all sequenced teneurins, and most predicted teneurins, lies a region with striking amino acid similarity to the C-terminal GHH toxin domain of certain prokaryotic YD repeat-containing proteins (Zhang et al., 2012; Ferralli et al., 2018). GHH toxins are prokaryotic nucleases that are predicted to be encapsulated in a YD repeat barrel (like the toxic C-terminal domain of the YD repeat-containing protein from *Y. entomophaga*). Though similar, the GHH toxin domain of teneurins lack the key glycine-histidine-histidine motif that is necessary for the bacterial enzyme’s nuclease activity (Zhang et al., 2012). However, when the GHH toxin domains of teneurin-1 or teneurin-2 are expressed in HEK 293 cells in culture, or when nanomolar concentrations of the purified GHH toxin domains of chicken teneurin-1 or chicken teneurin-2 are added to the culture medium, the cells rapidly undergo apoptosis (Ferralli et al., 2018). The toxicity may be related to nuclease activity, as purified GHH toxins from teneurin-1 and teneurin-2 cleave plasmid DNA and completely hydrolyze mitochondrial DNA *in vitro* (Ferralli et al., 2018).

The C-terminal 40 or 41 amino acids of teneurins is known as TCAP (from “teneurin C-terminal associated peptide”). The TCAP sequence was first identified by researchers who noted its similarity to corticotropin-releasing factor (Qian et al., 2004), and purified TCAP has profound effects on animal behavior when injected into brain ventricles (Tan et al., 2008). For example, TCAP-treated rats behave in acoustic startle, open field and elevated plus maze tests in a manner that is consistent with elevated anxiety. These and other remarkable studies with TCAP have recently been reviewed by others (Woelfle et al., 2016). The TCAP sequence partially overlaps with the GHH toxin domain and extends to the very C-terminus of the protein (see above). Interestingly, teneurins are known to bind to the G-protein coupled receptor latrophilin (Silva et al., 2011), and the teneurin domain responsible for this interaction is the TCAP (Woelfle et al., 2015). This may contribute to the localization of some teneurins, and the C-terminal toxin/TCAP domain, to developing synapses (Li et al., 2018).

### Teneurin Tertiary Organization

**FIGURE 2:**
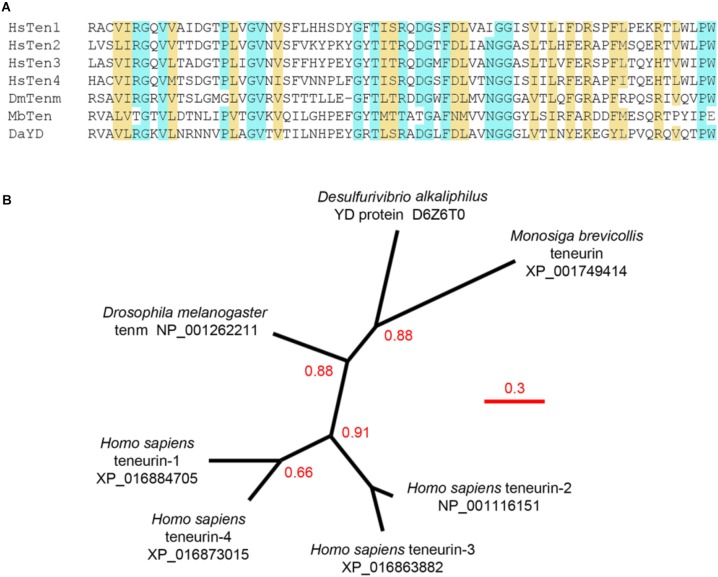
Analysis of the carboxypeptidase-like domain. **(A)** The carboxypeptidase-like domains from various teneurins (HsTen1-4, *Homo sapiens* teneurin-1 to 4; DmTenm, *Drosophila melanogaster* ten-m; MbTem, *Monosiga brevicollis* teneurin) and the *Desulfurivibrio alkaliphilus* YD protein (DaYD) aligned showing identity (blue) and strongly similar properties (yellow; >0.5 in the Gonnet PAM250 matrix). **(B)** An unrooted phylogenetic tree constructed using phylogeny.fr default parameters, TreeDyn and 100 rounds of bootstrapping. Branch support higher than 0.50 is indicated. Bacterial and choanoflagellate sequences segregate to the same clade, supporting the hypothesis that teneurins evolved through horizontal gene transfer. Teneurin-1 and teneurin-4, and teneurin-2 and teneurin-3, appear to have evolved through recent gene duplication events. Relevant UniProt ID and GenBank accession numbers are indicated. Scale bar = substitutions/site.

The stick diagrams used for describing the domain organization of teneurins can now be refined thanks to the pioneering X-ray crystallography done with a related bacterial protein (Busby et al., 2013) and the elegant X-ray crystallography and cryoelectron microscopy done with the extracellular domains of teneurins themselves (Jackson et al., 2018; Li et al., 2018). We now know that the region found between the EGF-like domains and the 6-bladed beta-propeller folds into a beta-sandwich domain that is reminiscent of either a fibronectin type III (FN3) repeat (Jackson et al., 2018) or an immunoglobulin (Ig)-like domain (Li et al., 2018), and this domain “plugs” the N-terminal end of the hollow YD barrel (Figure 1B). This FN3/Ig-like domain has two sub-regions, one of which is highly conserved across phyla and is rich in cysteines (Tucker et al., 2012), and another which is predicted by standard domain architecture software programs (e.g., Superfamily) to be a carboxypeptidase domain. The latter is particularly interesting because some bacterial YD proteins with a 6-bladed beta-propeller also have a carboxypeptidase domain in this region, and these amino acid sequences align with nearly 50% similarity with the same region in human teneurins (Figure 2A). This striking phylogenetic conservation suggests that this unstudied domain may be more than a plug: perhaps it is involved in proteolytic processing of teneurins or teneurin-associated proteins. The RHS core protein fits into the C-terminal end of the teneurin YD barrel, but interestingly, both recent studies of teneurin structure (Jackson et al., 2018; Li et al., 2018) showed the GHH toxin/TCAP domain poking out through the side of the barrel instead of being contained within the barrel like the toxins of bacterial YD proteins. Thus, the conformational changes that are believed to release the toxin from the YD barrel of prokaryotes may not be necessary for the release of the toxin/TCAP domain from teneurins. Moreover, the arrangement of the C-terminal region of teneurins revealed by cryoelectron microscopy means that the TCAP domain is available to bind to latrophilin without any prior processing or the disruption of the YD barrel. Perhaps the toxic nuclease near the C-terminus of teneurins can be released by regulated proteolytic activity after the teneurin reaches the cell membrane. Supporting this hypothesis is the observation that almost all teneurins examined to date have the conserved basic motif RxRR between the RHS core protein and the GHH toxin domain (Tucker et al., 2012), and similar motifs are known to be targeted by proteases that act extracellularly [e.g., by members of the proprotein convertase subtilisin/kexin family of peptidases (Rawlings, 2009)]. Future studies are needed to address this important aspect of teneurin biology.

### Differences Between the Teneurins

The overall domain organization of the four chordate teneurins is identical, but upon closer examination certain distinguishing features can be recognized (Figure 1C). For example, the intracellular domains of human teneurin-1 and teneurin-4 have both proline-rich SH3-binding domains and predicted nuclear localization sequences, but the intracellular domain of human teneurin-3 lacks SH3-binding prolines and the intracellular domain of teneurin-2 lacks a nuclear localization sequence. These differences, however, are not conserved across species. In the chicken, where teneurins have been widely studied, the intracellular domains of all four teneurins have predicted nuclear localization sequences, whereas in the mouse only teneurins-1 and -3 have predicted nuclear localization sequences (Tucker et al., 2012). Though the intracellular domains of chordate teneurins and the teneurins found in ecdysozoa share little sequence homology, the intracellular domains of both ten-a and ten-m from *D. melanogaster* and ten-1 from *C. elegans* have predicted nuclear localization sequences and SH3-binding domains (Tucker et al., 2012). However, whenever discussing teneurin nuclear localization sequences it is important to remember that the vast majority are only predicted *in silico*. The only experimental evidence that the intracellular domains of teneurins can be transported to the nucleus come from studies with chicken sequences in cell lines (Bagutti et al., 2003; Nunes et al., 2005), in chicken embryos (Kenzelmann et al., 2008; Kenzelmann Broz et al., 2010) and in *C. elegans* (Drabikowski et al., 2005).

In chordates a cysteine in the second teneurin EGF-like domain has been replaced by a tyrosine, and the cysteine in the fifth EGF-like domain has been replaced by a tyrosine (teneurin-2 and teneurin-3) or by a phenylalanine or tyrosine (teneurin-1 and teneurin-4). This general arrangement is also found in the teneurins of ecdysozoa, but not teneurins from lophotrochozoa. For example, the predicted teneurin from the blood fluke *Schistosoma mansoni* has only four EGF-like domains, and all have a complete complement of cysteines (Tucker et al., 2012). Thus, while the dimerization of teneurins via their EGF-like domains is widespread, in some animals teneurins may act as monomers.

Teneurin-2 and teneurin-3 from chordates, as well as the teneurins from almost all invertebrates, have a predicted furin cleavage site between the transmembrane domain and the EGF-like domains. This site was shown to be functional in teneurin-2 (Rubin et al., 1999; Vysokov et al., 2016), and its widespread phylogenetic conservation suggests that it is important for teneurin function. This processing would suggest that the extracellular domain of teneurins is shed from the cell surface. However, the extracellular domain appears to remain anchored to the remaining transmembrane part of the teneurin through non-covalent interactions (Vysokov et al., 2016). Whether or not such interactions are found in other teneurins with the predicted furin cleavage site remains to be determined.

Functional differences in the extracellular domains of different teneurins have also been identified. As mentioned earlier, homotypic interactions between the beta-propellers of teneurins are stronger than heterotypic interactions (Beckmann et al., 2013), suggesting that the beta-propellers have properties that are unique to different teneurin forms. Moreover, the C-terminal regions of different teneurins have different affinities for latrophilins (Boucard et al., 2014). These observations will likely be keys to our understanding of why teneurins have duplicated to become a multigene family independently in arthropods and in chordates (Tucker et al., 2012).

## The Evolution of Teneurins

An examination of sequenced metazoan genomes revealed that teneurins are found in all animals with a central nervous system, but not in sponges, *Trichoplax* or cnidarians (Tucker et al., 2012). Given the prominent expression of teneurins in the developing central nervous system of flies, worms and chordates, this led, at least temporarily, to the assumption that teneurins evolved together with a complex nervous system. However, when predicted proteins with the teneurin domain organization were searched for in non-metazoan sequences, a teneurin was discovered in the genome of the single-celled choanoflagellate *Monosiga brevicollis*. The teneurin from this choanoflagellate is remarkable in many ways. First, its domain organization matches that of chordate teneurins almost perfectly: it has an intracellular domain with a predicted nuclear localization sequence and an extracellular domain with eight EGF-like domains (all with six cysteine residues), a cysteine-rich domain and a carboxypeptidase domain, a beta-propeller, YD repeats and an RHS core domain (Figure 1D). It only lacks predicted furin cleavage sites found in most metazoan teneurins and a C-terminal GHH toxin/TCAP domain. Second, it is encoded on only four exons, the third of which contains 6829 residues and encodes almost all of the extracellular domain. Finally, BLAST searches of the sequences encoded on the third exon revealed that the extracellular domain of choanoflagellate teneurin was more similar to the YD proteins of bacteria than to the extracellular domain of metazoan teneurins. This pointed to the possibility that teneurins evolved via horizontal gene transfer from a bacterial prey (with a YD protein gene encoded on a single exon) to a single-celled predator prior to the evolution of metazoa from a choanoflagellate-like ancestor (Tucker et al., 2012).

Horizontal gene transfer into choanoflagellates from their prey (bacteria, algae, and diatoms) is well-documented, and many of these events have contributed genes that are still used in modern choanoflagellates, sometimes replacing similar host genes, and sometimes contributing novel enzymes that can be used by the host to exploit nutrient-deficient niches (Tucker, 2013). However, relatively few metazoan genes appear to have originated from a choanoflagellate gene that was in turn acquired from bacteria or algae. One survey revealed only two: dihydroxy-acid dehydratase and teneurins (Tucker, 2013).

As mentioned earlier, the extracellular domains of teneurins are remarkably similar to many prokaryotic YD proteins, and a protein similar to the modern-day YD proteins of bacteria is the most likely candidate for the ancestral teneurin. Some of these are illustrated schematically in Figure 1D. The carboxypeptidase-like domain found near the 6-bladed beta-propeller of all teneurins is also found in the YD protein of *Desulfurivibrio alkaliphilus*, an anaerobic, gram-negative, non-motile bacterium that lives in the extreme high-saline and high-pH soda lakes of North Africa (Melton et al., 2016). This YD protein has an uncharacterized toxin domain, but otherwise resembles, from the carboxypeptidase-like domain to the RHS core protein domain, the extracellular domain of teneurins. The remarkable similarity of the carboxypeptidase-like domain from the *D. alkaliphilus* YD protein and the similar domain of various teneurins is shown in Figure 2A. This stretch of approximately 70 amino acids is also particularly well-suited for establishing the phylogenetic relationships between the YD proteins and teneurins as well as teneurins themselves (Figure 2B). Consistent with a proposed origin from bacteria, the choanoflagellate teneurin and the bacterial sequence are found in the same clade. In chordates teneurin-1 and teneurin-4 are likely to have evolved through gene duplication, as have teneurin-2 and teneurin-3. These paired relationships are consistent with the organization of the intracellular domains, the predicted extracellular domain furin cleavage sites, and the residues that replace the cysteine residues in the EGF-like domains. Analysis of other domains results in similar phylogenetic trees (Tucker et al., 2012).

From studies of teneurin evolution we can gain insight into teneurin function. For example, what is known about the function of the YD repeat-containing proteins of bacteria? First and foremost, they are toxins (Zhang et al., 2012). The C-terminal toxin domain is encased in a YD repeat barrel, apparently to protect the cell that is expressing the YD repeat protein from the toxin (Busby et al., 2013). One class of bacterial toxins with YD barrels are the ABC toxins (ffrench-Constant and Waterfield, 2005). The B and C parts of the toxin are expressed either from a single gene or on two or more adjacent genes, and they can form a complex containing a beta-propeller, YD repeats, RHS core protein domain and C-terminal toxin domain. Multiple versions of the C gene allow different types of toxins to be deployed (Figure 1D). The A protein provides a way for the toxin to get into the cell, either by making a pore or by inserting into the membrane and acting as a receptor for the BC component (Busby et al., 2013). Other YD proteins are, like teneurins, type-2 transmembrane proteins. They appear to be members of the “toxin on a stick” type of bacterial polymorphic toxins (Jamet and Nassif, 2015). Polymorphic toxins are part of self-recognition between bacteria and are used in interbacterial warfare; when identical proteins interact they do not release the C-terminal toxin domain, but when dissimilar proteins interact the toxin domains are released. Given the complementary patterns of expression of many teneurins during the development of the nervous system (see below), one can hypothesize that heterotypic interactions between teneurins may somehow result in the release of the GHH toxin domain. This in turn could lead to programmed cell death or the pruning of dendrites. However, to date this inviting hypothesis is only supported by circumstantial evidence.

## Patterns of Teneurin Expression

### *Drosophila melanogaster* and *Caenorhabditis elegans*

The first description of teneurin expression came from Baumgartner and Chiquet-Ehrismann (1993), who used *in situ* hybridization to determine the sites of expression of ten-a in *Drosophila*. They found widespread ten-a expression in the early embryo and high levels of expression in the developing ventral nerve cord following germ band retraction. Ten-a transcripts are also observed in muscle apodemes, the clypeolabrum and the antenna-maxillary complex. This work was followed by two independent reports (Baumgartner et al., 1994; Levine et al., 1994) describing the expression of ten-m. Both papers reported ten-m expression in seven stripes during the blastoderm and germ band extension stages of development, and the eventual expression of ten-m in the ventral nerve cord and in cardioblasts. Both papers go on to describe the failure of ventral denticle belts to fuse in P-element insertion mutants (i.e., an “oddless” pair rule phenotype), and one illustrates the disruption of the central nervous system in these mutants (Levine et al., 1994). Higher resolution studies using LacZ expression under the ten-m promoter revealed expression in imaginal disks (Levine et al., 1997; Minet et al., 1999). These studies reveal ten-m expression in sensory mother cells and the R7 photoreceptor in developing ommatidia. The expression of teneurins in cardioblasts was revisited and expanded on by Volk et al. (2014). Ten-a is expressed at the border of cardioblasts and pericardial cells, and ten-m is expressed by both cardioblasts and pericardial cells. However, ten-m and ten-a mutants do not have heart defects (Volk et al., 2014).

**FIGURE 3:**
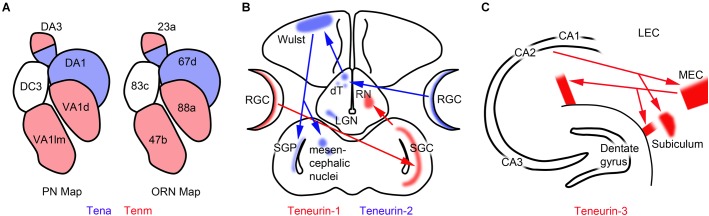
Teneurins are expressed by interconnected populations of neurons. **(A)** In *Drosophila*, olfactory receptor neurons (ORNs) expressing ten-a synapse in antennal lobe glomeruli with projection neurons (PN) expressing ten-a. Ten-m expressing neurons also synapse together in the antennal lobe. **(B)** In the developing chicken, teneurin-1 is expressed in the tectofugal visual pathway, whereas teneurin-2 is expressed in the thalamofugal visual pathway. dT, dorsal thalamic nuclei; LGN, lateral geniculate nucleus; RGCs, retinal ganglion cells; RN, rotund nucleus; SGC, stratum griseum centrale; SGP, stratum griseum periventriculare. **(C)** In the mouse hippocampus teneurin-3 is expressed in the CA1 region, the subiculum and the medial entorhinal cortex (MEC). Tracer studies show that these regions are connected to each other. LEC, lateral entorhinal cortex. The data summarized in this figure were published by Rubin et al. (2002); Hong et al. (2012), and Berns et al. (2018), respectively.

The developing olfactory system of *Drosophila* has proven to be a particularly useful model for studying both the expression of teneurins and their roles in development. In *Drosophila*, olfactory receptor neurons (ORNs) are the primary neurons that receive olfactory information. ORN axons synapse with the dendrites of projection neurons (PNs) in glomeruli found in the antennal lobe, and the PNs in turn send their axons elsewhere in the central nervous system. ORN/PN pairs have been mapped precisely. For example, Or47b ORNs normally project to the VA 1 lm glomerulus, and Mz19 PN dendrites are found in an adjacent glomerulus. Hong et al. (2012) used this model to screen for genes that might regulate the development of precise neural networks. They observed that overexpression of ten-m in the Mz19 PNs leads to abnormal connections between Mz19 neurons and Or47b ORNs. A similar system was used for a second screen: Or88a ORNs normally project to the VA1d glomerulus where they intermingle with Mz19 PN dendrites. Overexpression of ten-a in Mz19 PNs disrupts the normal intermingling of Mz19 and Or88a dendrites. In a screen of 410 candidate genes, only the ectopic misexpression of ten-a and ten-m caused these disruptions. High levels of ten-a and ten-m are found in antennal lobe glomeruli in mostly non-overlapping patterns, but both are found in low levels in all glomeruli (Hong et al., 2012). In five glomeruli examined in detail, ORNs expressing high levels of ten-m send axons to glomeruli with PNs expressing high levels of ten-m, and the axons of ORNs expressing high levels of ten-a are found in glomeruli with PN dendrites that also express high levels of ten-a (Figure 3A). Genetic and RNAi knockdowns result in shifting patterns of ORN/PN interactions, indicating that homophilic interactions between the teneurins are necessary for proper synaptic patterning.

*Drosophila* is also a useful model for studying the roles of teneurins in the development of neuromuscular junctions. In this system ten-a is expressed by neurons and is presynaptic, while most of the ten-m is expressed by muscle and is post-synaptic (Mosca et al., 2012). *In vivo* ten-a and ten-m appear to form a complex, and disruption of the expression of either or both leads to severe defects in the neuromuscular junction. These defects include disorganization of microtubules presynaptically, and disruption of alpha-spectrin post-synaptically. Heterophilic interactions between the ten-a and ten-m expressed at basal levels in antennal lobe glomeruli also appear to occur in neuronal synapses (Mosca and Luo, 2014). Presynaptic ten-a controls the number of ORN synapses that are found in a glomerulus, and ten-a/ten-m interactions regulate presynaptic active zone number. The possible roles of teneurins in synaptogenesis were reviewed by Mosca (2015).

There is a single teneurin gene in *C. elegans*, but two transcripts are generated via alternate promoters (Drabikowski et al., 2005). The differences between the variants lie in the size of the intracellular domain: Ten-1L has a longer intracellular domain that includes two predicted nuclear localization sequences, while Ten-1S has a severely truncated intracellular domain. The extracellular domain of the variants is identical. Ten-1L expression was studied using a GFP translational fusion protein (Drabikowski et al., 2005). It is found in neurons in the ventral nerve cord and in the pharyngeal nerve ring. There is also significant non-neuronal expression in vulvar and diagonal muscles, gonadal distal tips cells and in the vas deferens, among other sites (Table 1). After injection with Ten-1 RNAi there are neuronal pathfinding defects, abnormal gonad development, and severe morphological defects resulting from abnormal migration of hypodermal cells. Similar defects are seen in Ten-1 null mutants (Drabikowski et al., 2005). In the mutant Ten-1(et5), which has a point mutation leading to a premature stop codon near the end of the EGF-like domains, defects resulting from stalled growth cone migration and abnormal pathways of neurite outgrowth are seen in the pharyngeal nerve ring (Mörck et al., 2010). Unlike Ten-1 null mutants, Ten-1(et5) worms typical live to become reproductive adults, suggesting that the intracellular domain and EGF-like domains can impart some survival benefit. Ten-1 may also play a role in the organization of the extracellular matrix, as basement membrane integrity is compromised in Ten-1 null larvae (Trzebiatowska et al., 2008).

**Table 1:** Non-neuronal expression of teneurins^∗^.

Species	Teneurin	Stage	Tissue and references
*D. melanogaster* (fruit fly)	ten-a	Embryo	Antenna-maxillary complex (Baumgartner and Chiquet-Ehrismann, 1993)
			Muscle apodeme (Baumgartner and Chiquet-Ehrismann, 1993)
			Clypeolabrum (Baumgartner and Chiquet-Ehrismann, 1993)
			Cardioblasts (Volk et al., 2014)
		Larva	Muscle (Mosca et al., 2012)
		ten-m	Embryo blastoderm stripes (Baumgartner et al., 1994; Levine et al., 1994)
			Cardioblasts (Baumgartner et al., 1994; Levine et al., 1994; Volk et al., 2014)
			Lymph gland (Baumgartner et al., 1994)
			Tracheal system (Baumgartner et al., 1994)
		Larva	Imaginal disks (Levine et al., 1997; Minet et al., 1999)
			Muscle (Mosca et al., 2012)
*C. eleeans* (nematode)	ten-1	1.5 fold	Precursors of gut, somatic gonad, pharynx (Drabikowski et al., 2005)
		L4	Distal tip cells (Drabikowski et al., 2005)
		L4, adult	Vulva muscles (Drabikowski et al., 2005)
		Adult	Diagonal muscles (Drabikowski et al., 2005)
			Coelomocytes (Drabikowski et al., 2005)
			Vas deferens (Drabikowski et al., 2005)
*D. rerio* (zebrafish)	Teneurin-3	10, 14 hpf	Notochord (Mieda et al., 1999)
		17 hpf	Somites (Mieda et al., 1999)
		20, 36 hpf	Branchial arches (Mieda et al., 1999)
		36 hpf	Fin buds (Mieda et al., 1999)
*G. gallus* (chicken)	Teneurin-1	Stage 23	Dorsal limb ectoderm (Tucker et al., 2001)
			Ventral limb mesenchyme (Tucker et al., 2001)
	Teneurin-2	Stage 19/20	Distal limb bud (Tucker et al., 2001)
		Stage 21	Branchial arch mesenchyme (Tucker et al., 2001)
			Heart (Tucker et al., 2001)
			Flank mesoderm (Tucker et al., 2001)
			Notochord (Tucker et al., 2001)
			Somites (Tucker et al., 2001)
			Lens capsule (Tucker et al., 2001)
		Stages 21, 23	Apical ectodermal ridge (Tucker et al., 2001; Kenzelmann Broz et al., 2010)
		Stages 21, 27	Craniofacial mesenchyme (Tucker et al., 2001)
		Stages 26, 27	Distal and proximal limb mesenchyme (Tucker et al., 2001)
	Teneurin-3	Stage 23	Dorsal limb mesenchyme (Kenzelmann Broz et al., 2010)
	Teneurin-4	Stages 20, 21	Apical ectodermal ridge (Tucker et al., 2000; Kenzelmann Broz et al., 2010)
		Stages 20, 21	Zone of polarizing activity (Tucker et al., 2000)
		Stage 21	Gut mesenchyme (Kenzelmann Broz et al., 2010)
			Basement membranes (Kenzelmann Broz et al., 2010)
		Stages 21, 24	Branchial arches (Tucker et al., 2000; Kenzelmann Broz et al., 2010)
		Stages 23, 24	Anterodistal limb bud mesenchyme (Kenzelmann Broz et al., 2010)
		Stages 23, 30	Periocular mesenchyme (Kenzelmann Broz et al., 2010)
		Stages 23, 36	Lung mesenchyme (Kenzelmann Broz et al., 2010)
			Proximal limb mesenchyme (Kenzelmann Broz et al., 2010)
		Stage 43	Intestinal muscularis mucosa (Kenzelmann Broz et al., 2010)
			Atrioventricular valves (Kenzelmann Broz et al., 2010)
			Epicardium (Kenzelmann Broz et al., 2010)
*M. musculus* (mouse)	Teneurin-1	Adult	Corneal epithelium (Oohashi et al., 1999)
	Teneurin-3	e7.5	Neural plate and neural folds (Zhou et al., 2003)
		e8.5, 10.5	Branchial arches (Zhou et al., 2003)
		e9.5, 10.5	Anterior somites (Zhou et al., 2003)
			Limb buds (Zhou et al., 2003)
	Teneurin-4	e7.5	Neural plate and neural folds (Zhou et al., 2003)
		e8.5, 9.5, 10.5	Posterior somites (Zhou et al., 2003)
		el0.5	Branchial arches (Zhou et al., 2003)
			Periocular region (Zhou et al., 2003)
		e13.5, 18.5	Cartilage (Suzuki et al., 2014)
		P6	Oliaodendrocvtes (Suzuki et al., 2012)
*R. norvegicus* (rat)	Teneurin-2	e20, P0	Odontoblasts^†^ (Torres-da-Silva et al., 2017)


*^∗^Expression determined by *in situ* hybridization, immunohistochemistry, or both. ^†^Similar results are also reported in this paper using human tissues.*

### Zebrafish

One of the first detailed descriptions of teneurin expression in a vertebrate was reported by Mieda et al. (1999), who cloned and sequenced zebrafish teneurin-3 and teneurin-4 while searching for genes that were regulated by Islet-3. Using *in situ* hybridization they showed that teneurin-3 is transiently expressed in the notochord, somites, branchial arches, and central nervous system (Table 1). Teneurin-4 is expressed faintly during gastrulation, and after that is primarily expressed in the developing brain. Within the central nervous system, teneurin-3 and teneurin-4 are found in largely complementary patterns. For example, at 23 hpf teneurin-4 is found in two lines that wrap around the rostral diencephalon and teneurin-3 is expressed in the region between the lines. The sharp borders between the domains expressing these two teneurins become less clear later in development. The expression of teneurin-4 in narrow bands of cells in the zebrafish central nervous system is remarkably similar to the first report of teneurin-4 expression in the mouse: in E10.5 and E11.5 mouse embryos, teneurin-4 transcripts are found in a sharp line at the boundary between the midbrain and hindbrain (Wang et al., 1998).

Teneurin-3 is also found in the developing zebrafish retina (Antinucci et al., 2013). It is expressed by retinal ganglion cells and amacrine cells, which synapse with each other in the inner plexiform layer. Teneurin-3 is also expressed by the targets of retinal ganglion cell projections in the tectum. Knockdown of teneurin-3 expression with antisense morpholino oligonucleotides leads to both abnormal arborization of retinal ganglion cells in the inner plexiform layer and to abnormal pathfinding in the tectum (Antinucci et al., 2013). Zebrafish larvae normally adapt their level of pigmentation to background lighting and appear lighter in bright light and darker at lower levels of illumination. The teneurin-3 morphants are darkly pigmented in bright light, indicating that they probably have severe visual deficits. A teneurin-3 knockout zebrafish was also generated by TALEN genome editing (Antinucci et al., 2016). In the teneurin-3 knockouts amacrine cells that normally express teneurin-3 fail to arborize in the appropriate strata of the inner plexiform layer. The authors of this study go on to show that teneurin-3-expressing neurons form a distinctive circuit in the zebrafish retina that is responsible for orientation selectivity.

### Patterns of Expression in the Visual Systems of Birds and Mice

The first description of teneurin-1 features low-resolution *in situ* hybridization images pointing to developing neurons as a primary site of expression (Minet et al., 1999). In the developing chicken diencephalon teneurin-1 transcripts are found in the rotund nucleus, and in the optic tectum teneurin-1 is expressed by the large neurons of the stratum griseum centrale. This study was followed by a paper that compared the expression of teneurin-1 and teneurin-2 in the developing avian central nervous system (Rubin et al., 1999). Teneurin-1 and teneurin-2 mRNAs are both found in the developing thalamus, but in different nuclei, and in the optic tectum teneurin-2 expression was observed in layers that straddled the stratum griseum centrale but is missing from the stratum griseum centrale itself. This led to the conclusion that teneurin-1 and teneurin-2 are expressed in different populations of developing neurons. Similar results were reported in a study of the developing mouse that included other teneurin forms (Zhou et al., 2003), but the complementary patterns are not as obvious in the mouse as in the chicken. More detailed mapping of expression using antibodies specific for teneurin-2 led to the remarkable observation that teneurin-1 and teneurin-2 are each expressed by interconnected populations of neurons (Rubin et al., 2002). This is particularly clear in the developing visual system of the chicken, where teneurin-1 is expressed in the developing tectofugal visual pathway, and teneurin-2 is expressed in the developing thalamofugal visual pathway (Figure 3B). The timing of expression typically follows the period of growth cone pioneering and neurite outgrowth and coincides with periods of synaptogenesis, pruning, and apoptosis.

In the mouse, most retinal ganglion cells project to the lateral geniculate nucleus or the superior colliculus. The former projections form a critical map of visual field information prior to further processing in the cortex. Retinal ganglion cells also make a map of the visual field in the superior colliculus, and this map is important for integrating responses to auditory, somatosensory, and visual information (Kandel et al., 2012). Appropriate binocular vision requires that retinal ganglion cells from each retina project to either the ipsilateral or contralateral superior colliculus and lateral geniculate nucleus. Teneurins appear to be critical for the successful development of these visual circuits. Using *in situ* hybridization, Leamey et al. (2007) found that teneurin-3 is expressed in a gradient in the developing mouse retinal ganglion cell layer, with highest levels of expression in the ventral retina. This was confirmed with quantitative PCR. Teneurin-3 is also expressed in a gradient within the lateral geniculate nucleus, with highest levels of expression found in the dorsal part of the nucleus. As retinal ganglion cells from the ventral retina project to the dorsal part of the lateral geniculate nucleus, the authors next chose to study these projections in teneurin-3 knockout mice. The brains and retinas of the knockout mice appeared normal in standard histological preparations. However, a tracing study with the knockouts reveals abnormal ipsilateral projections that are no longer limited to the dorsal part of the lateral geniculate nucleus, as well as abnormal connections between the lateral geniculate nucleus and the visual cortex (Leamey et al., 2007; Merlin et al., 2013; for review see Leamey and Sawatari, 2014). Behavioral studies are consistent with the hypothesis that teneurin-3 knockout mice lack binocular vision (Leamey et al., 2007). Teneurin-3 mRNA is also expressed in a gradient in the superior colliculus, with highest expression medially and the lowest laterally (Dharmaratne et al., 2012). This also corresponds to the high ventral, low dorsal expression of teneurin-3 in the retina. When the teneurin-3 knockout mice were further analyzed, ipsilateral projections to the superior colliculus are highly abnormal, just as they are in the lateral geniculate nucleus. EphA7 is significantly reduced, and EphB1 is significantly upregulated, in the visual system of teneurin-3 knockout mice. This suggests that teneurins may work together with ephrin/Eph signaling in this system (Glendining et al., 2017). Other teneurins may also be critical for the development of visual pathways. Like teneurin-3, teneurin-2 is expressed by interconnected populations of neurons in the murine retina, lateral geniculate nucleus and superior colliculus (Young et al., 2013), and in teneurin-2 knockout mice there is a reduction in the number of retinal ganglion cells that project to ipsilateral targets. Interestingly, antibodies to teneurin-4 label retinal ganglion cell axons in the nasal, but not temporal, retina of the chicken embryo (Kenzelmann Broz et al., 2010). This may indicate that other teneurins may regulate the development of other circuits in the visual system.

### Expression in the Thalamus, Cortex, and Hippocampus

The thalamus contains dozens of nuclei that generally act as relay stations between sensory inputs and the cerebral cortex. The first studies of teneurin-1 and teneurin-2 noted that these teneurins are prominently expressed in distinct populations of thalamic nuclei (Minet et al., 1999; Rubin et al., 1999), some of which are interconnected parts of the visual system (Rubin et al., 2002). The importance of normal teneurin-2 and teneurin-3 expression in the lateral geniculate nucleus, which is found in the thalamus, was described in the preceding section.

In order to learn more about the repertoire of guidance molecules that are responsible for establishing the complicated set thalamic of circuits, Bibollet-Bahena et al. (2017) performed *in situ* hybridization with teneurin probes on sections through the rostral and caudal thalamic nuclei of embryonic and newborn mouse brains. Each teneurin has a distinctive pattern of expression. There is significant overlap between the expression patterns of teneurin-2, teneurin-3 and teneurin-4, with teneurin-1 forming a pattern that is largely complementary to the other teneurins. For example, in the rostral thalamus teneurin-1 is expressed in dorsal thalamic nuclei and the reticular nucleus, and these regions show little or no expression of the other teneurins. The other teneurins, but not teneurin-1, are expressed in the laterodorsal nucleus, and both teneurin-2 and teneurin-3 are expressed in the ventral anterior nucleus.

One of the thalamic nuclei, the parafascicular nucleus, projects to the striatum. Teneurin-3 is expressed in a dorsal to ventral gradient in both the parafascicular nucleus and the striatum (Tran et al., 2015). As neurons in the dorsal parafascicular nucleus project to the dorsal striatum, this gradient of teneurin-3 expression matches earlier studies of retinal projections to the lateral geniculate nucleus and superior colliculus (Leamey et al., 2007; Dharmaratne et al., 2012). The size of these regions and the numbers of neurons found in them are similar in both wild type and teneurin-3 knockout mice, but anterograde tracer studies revealed abnormal projections to the striatum as well as the loss of distinctive cluster terminals within the striatum (Tran et al., 2015). Consistent with the known functions of the parafascicular nucleus and striatum in goal-directed learning, teneurin-3 knockout mice exhibit delayed acquisition of motor skills (Tran et al., 2015).

The cerebral cortex is patterned both by intrinsic factors originating in neuronal progenitors and by extrinsic factors that originate in thalamocortical projections. One of the key factors regulating the intrinsic patterning of the neocortex is the homeobox transcriptional regulator EMX2, which is expressed in a high-caudal to low-rostral gradient in the cortical plate. Teneurin-4 was identified in a screen of genes that are differentially regulated in the Emx2(-/-) mouse (Li et al., 2006). In the developing mouse brain, teneurin-4 is normally expressed by cortical neurons and their precursors in a gradient that matches that of EMX2. In the Emx2(-/-) mouse, there is both a reduction in the overall level of expression of teneurin-4 and a loss of the expression gradient (Li et al., 2006). There is also strong evidence that teneurin-1 expression in the cortex is regulated by EMX2 (Beckmann et al., 2011). Finally, teneurin-3 was also identified as a gene that is differentially regulated in the developing mouse cortex (Leamey et al., 2008). It is highly expressed in layer V of the caudal-most cortex, which corresponds well with its prominent roles in the patterning of visual system (see above). Interestingly, overexpression of teneurin-3 in the embryonic cerebral cortex via in utero electroporation leads to clustering of teneurin-3-expressing cells, suggesting stronger homophilic interactions between these neurons when compared with their neighbors.

The first study of teneurin expression in the mouse using immunohistochemistry described teneurin-1 in the molecular layer of the CA3 region of the adult hippocampus as well as the molecular layer of the cerebellum (Oohashi et al., 1999). This pioneering work was followed with a comparative study showing the expression of all four teneurins in the adult hippocampus: teneurin-1 is expressed in CA 3 and the dentate gyrus, teneurin-2 is expressed most strongly in the CA 1 and CA 2 regions, teneurin-3 is limited to the stratum lacunosum moleculare, and teneurin-4 is most prominently expressed in the molecular layer of the dentate gyrus and in the stratum lacunosum moleculare and stratum oriens of the CA 3 region (Zhou et al., 2003).

A recent study addressed the importance of normal teneurin-3 expression in the developing hippocampus (Berns et al., 2018). Teneurin-3 is expressed by a patch of neurons in the proximal part of the CA1 region of the P10 hippocampus, as well as in the neighboring distal subiculum and the medial entorhinal cortex (MEC). Tracer injected into the MEC labels teneurin-3-positive neurons in the subiculum and the proximal CA1, and tracer injected in the lateral entorhinal cortex labeled both the proximal CA1 and the distal subiculum, demonstrating that the neurons expressing teneurin-3 form a neural network (Figure 3C). The model was then exploited experimentally with a teneurin-3 knockout mouse to show the necessity of normal teneurin-3 expression in the development of CA1/subiculum connections (Berns et al., 2018).

### Non-neuronal Patterns of Teneurin Expression in Birds and Mammals

**FIGURE 4:**
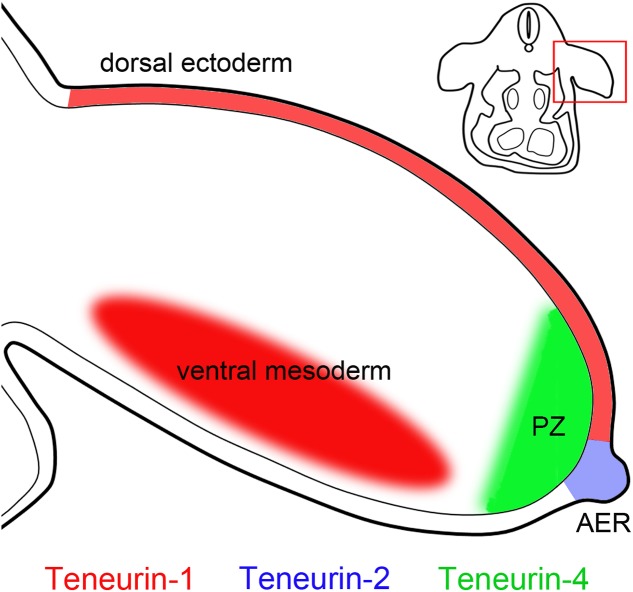
Teneurin expression in the developing limb. Teneurins show complementary patterns of expression in developing chicken limbs. For example, teneurin-2 is expressed in the apical ectodermal ridge (AER), teneurin-4 is expressed in the anterior part of the underlying progress zone (PZ), and teneurin-1 is expressed in the ectoderm dorsally, and in the mesenchyme ventrally (Tucker et al., 2000, 2001; Kenzelmann Broz et al., 2010). The inset shows a schematic cross section through a chicken embryo and the location of the developing limb.

While the name “teneurin” comes from “ten-m” and “neuron” (Minet et al., 1999), it is important to remember that teneurins are also expressed in many non-neuronal tissues. As described above, teneurins are expressed in stripes in *Drosophila* embryos (Baumgartner et al., 1994; Levine et al., 1994), by motile cells and muscles in *C. elegans* (Drabikowski et al., 2005) and in somites and branchial arches in zebrafish (Mieda et al., 1999). In early chicken embryos antibodies to teneurin-4 immunostain the mesenchyme in many areas and co-localize with laminin in or near basement membranes (Kenzelmann Broz et al., 2010). Developing limbs also show distinctive and temporally dynamic patterns of teneurin expression. The apical ectodermal ridge (AER) is a prominent site of teneurin-2 expression (Tucker et al., 2001; Kenzelmann Broz et al., 2010). The teneurin-4 is expression pattern is more dynamic. It is initially observed in both the AER and the zone of polarizing activity, but later it is seen in the distal mesenchyme underlying the AER on the anterior part of the limb (Tucker et al., 2000; Kenzelmann Broz et al., 2010). Teneurin-1 expression in the developing limb is particularly interesting. Antibodies to the intracellular domain of teneurin-1 stain the cell surface of ectodermal cells in the dorsal limb, but they stain the cell nucleus in mesenchyme in the ventral limb (Kenzelmann Broz et al., 2010). These patterns are summarized in Figure 4. A recent study (Pickering et al., 2017) found that teneurin expression changes when retinoic acid-soaked beads are applied to the anterior part of the limb bud (teneurin-4 expression decreases, while teneurin-2 expression increases), but the roles of teneurins in limb patterning are unknown. These and other patterns of teneurin expression in non-neuronal tissues are summarized in Table 1.

## Conclusion

Since their serendipitous discovery 25 years ago considerable progress has been made in our understanding of teneurin organization, evolution and expression. The intracellular domain is more variable than the rest of the protein, and its function remains mostly a mystery. In particular, its processing and localization to the nucleus at some, but not all, sites of expression is an observation in dire need of additional experimental work. More is known about the extracellular domain of teneurins, which apparently evolved from the extracellular domain of a prokaryotic YD protein via horizontal gene transfer. We now know that the YD repeats of both the prokaryotic YD proteins and the teneurins fold into a hollow barrel with a nearby beta-propeller that can be used as a protein–protein interaction domain. Remaining work to be done includes studies of the highly conserved carboxypeptidase-like domain and whether or not the C-terminal domain can be released to act as a toxin. And if so, what triggers its release? In the central nervous system of vertebrates and flies teneurins appear to be expressed in largely non-overlapping patterns than correspond to interconnected populations of neurons. As genetic manipulation of this pattern leads to disruption of the development of these networks, teneurins appear to be key players in brain development. However, just how teneurins accomplish this is unclear. Do they act primarily through differential adhesion, or is the more important interaction the one between TCAP and latrophilins? And is the GHH toxin domain somehow involved in this process? Finally, studies should not neglect the interesting sites of non-neuronal expression of teneurins, such as developing limbs. During the next quarter century, discovering the answers to these questions will present researchers with special challenges.

## Author Contributions

The author confirms being the sole contributor of this work and has approved it for publication.

## Conflict of Interest Statement

The author declares that the research was conducted in the absence of any commercial or financial relationships that could be construed as a potential conflict of interest.
